# Enhanced pro-apoptosis gene signature following the activation of TAp63α in oocytes upon γ irradiation

**DOI:** 10.1038/s41419-022-04659-2

**Published:** 2022-03-04

**Authors:** Niclas Fester, Elisabeth Zielonka, Jakob Goldmann, Ann-Sophie Frombach, Uta Müller-Kuller, Niklas Gutfreund, Kristina Riegel, Jos G. A. Smits, Enrico Schleiff, Krishnaraj Rajalingam, Huiqing Zhou, Stefan Simm, Volker Dötsch

**Affiliations:** 1 Institute of Molecular Biosciences, Goethe University, 60438 Frankfurt, Germany; 2 Institute of Biophysical Chemistry and Center for Biomolecular Magnetic Resonance, Goethe University, 60438 Frankfurt, Germany; 3 European Molecular Biology Laboratory (EMBL), 69117 Heidelberg, Germany; 4 Departments of Human Genetics, Radboud Institute of Molecular Life Sciences, Radboud University Nijmegen Medical Centre, Nijmegen, Netherlands; 5 Georg-Speyer Haus, Frankfurt, 60596 Germany; 6 Cell Biology Unit, University Medical Center Mainz, JGU-Mainz, 55131 Mainz, Germany; 7 Departments of Molecular Developmental Biology, Faculty of Science, Radboud University, Nijmegen, Netherlands; 8 Institute of Bioinformatics, University Medicine Greifswald, Greifswald, Germany

**Keywords:** Radiotherapy, Mechanisms of disease

## Abstract

Specialized surveillance mechanisms are essential to maintain the genetic integrity of germ cells, which are not only the source of all somatic cells but also of the germ cells of the next generation. DNA damage and chromosomal aberrations are, therefore, not only detrimental for the individual but affect the entire species. In oocytes, the surveillance of the structural integrity of the DNA is maintained by the p53 family member TAp63α. The TAp63α protein is highly expressed in a closed and inactive state and gets activated to the open conformation upon the detection of DNA damage, in particular DNA double-strand breaks. To understand the cellular response to DNA damage that leads to the TAp63α triggered oocyte death we have investigated the RNA transcriptome of oocytes following irradiation at different time points. The analysis shows enhanced expression of pro-apoptotic and typical p53 target genes such as CDKn1a or Mdm2, concomitant with the activation of TAp63α. While DNA repair genes are not upregulated, inflammation-related genes become transcribed when apoptosis is initiated by activation of STAT transcription factors. Furthermore, comparison with the transcriptional profile of the ΔNp63α isoform from other studies shows only a minimal overlap, suggesting distinct regulatory programs of different p63 isoforms.

## Introduction

The production of both female and male gametes is subject to tight quality control measures. Interestingly, the quality control mechanisms differ in both sexes [1–4]. p63 is the most important member of the p53 family involved in the surveillance of genetic quality in oocytes [5–8]. This function of p63 is most likely the original one of the entire family, while other functions such as tumor suppression have developed later [5]. This notion is supported by the observation that organisms like *C. elegans* express a p53-like protein in their germ cells [9, 10] that based on its domain structure is more p63-like than p53-like [11]. In mice, oocytes enter dictyate arrest between P2 and P5. During this dictyate arrest phase the longest p63 isoform, TAp63α, is highly expressed and retains its expression level until oocytes are recruited for ovulation [7].

We showed that during dictyate arrest TAp63α is kept in an inactive and only dimeric conformation [12]. Detection of DNA double-strand breaks results in the activation of the kinase ATM which further activates the kinase CHK2. CHK2 phosphorylates TAp63α on S582 [13] located in a loop between the SAM domain and the transactivation inhibitory domain [14, 15]. While this phosphorylation has no influence on the conformational state of TAp63α, it recruits another kinase, CK1 [16], which typically requires pre-phosphorylated substrates [17, 18]. We could show that CK1 adds four more phosphate groups in a sequential manner. Of these, the third one is the decisive phosphorylation event that results in the opening of the closed dimer into an open and tetrameric state [16]. Since this transition is irreversible [19], this third phosphorylation event constitutes “the point of no return”. This decisive third phosphorylation is the slowest, which enables the oocyte to set the level of DNA damage that triggers apoptosis [20]. While these investigations provide a detailed molecular picture of the switch that decides between life and death of the damaged oocytes, we know less about the processes taking place at the cellular level except that activation of TAp63α results in the expression of PUMA and NOXA [21]. As oocyte death is triggered also by DNA damaging chemotherapeutics leading to premature ovarian insufficiency (POI) in female cancer patients, understanding how the oocyte decides between initiating apoptosis and DNA repair is crucial for the development of a future oocyte preserving therapy. As a further step in this direction, we have investigated the development of the oocyte transcriptome in a time-dependent manner and correlated it with the activation kinetics of TAp63α.

## Results

### Kinetics of activation

**Fig. 1:**
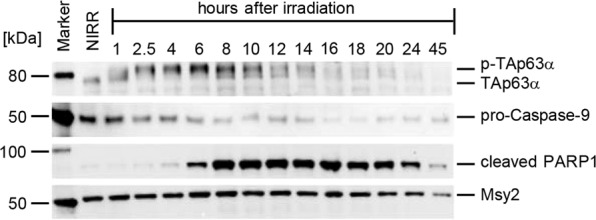
Time dependence of the level of several proteins in mouse oocytes following irradiation of ovaries with 0.5 Gy. Western blot analysis of the levels of TAp63α, its activated, phosphorylated form pTAp63α, pro-Caspase-9, cleaved PARP1, and Msy2 are shown. Msy2, a marker of oocytes, expressed in both primary as well as growing oocytes, was used as a control. The time traces indicate that after 2.5 h virtually all of TAp63α is converted to the activated form and after 6 h the signal of cleaved PARP1 starts to appear.

Recently, we had determined the kinetics of activation of TAp63α as well as the kinetics of apoptosis in mouse ovary culture by measuring the appearance of the signal of cleaved PARP and the decline of the volume of all primordial cells following the irradiation of ovaries with 0.5 Gy [20]. These investigations have shown that after 2.5 h virtually all of TAp63α has been converted to the full tetrameric state [20]. A strong cleaved PARP signal and a decrease of pro-caspase 9 can be detected at 6 h after irradiation (ref. [20], Fig. 1 and Supplementary Fig. 1). Consistently, as previously shown, the total volume of primary oocytes as measured by GCNA-positive cells in the ovary using immunofluorescence showed a strong decrease at 6 h [20]. Together these data suggest that apoptosis is fully initiated at the 6 h time point. We, therefore, decided to characterize the initial phase of the cellular response in which the decision about death or survival of the oocytes is taken.

### Major changes in gene expression occur after 2.5 h following γ-irradiation

To investigate the dynamics of gene expression changes following γ-irradiation, we performed transcriptome analysis by RNA-seq. After ovaries of P5 mice were γ-irradiated with 0.5 Gy, oocytes were isolated following standard protocols, to ensure that mRNAs originated only from oocytes. We prepared samples for time points of 1, 2.5, 4, and 6 h after γ-irradiation as well as before γ-irradiation (0 h) for comparison. For all samples between ~70 and ~80% of all reads could be mapped to the reference transcriptome of the mouse (Ensembl version 92) [22] leading to relative expression profiles represented by pseudo counts based on the mapper Salmon (Supplementary Table 1).

**Fig. 2:**
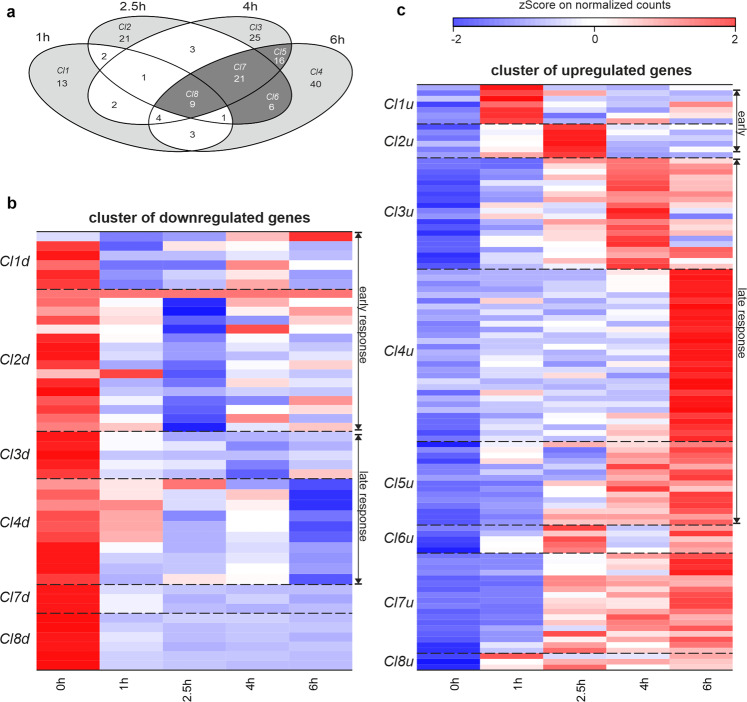
Differentially expressed genes in oocytes following γ-irradiation. **a** The Venn diagram shows the 167 protein-coding DEGs for the different time points (1, 2.5, 4, 6 h) compared to 0 h. Clusters (*Cl1*–*Cl8*) are split in up- and downregulated genes for visualization in (**b**) and (**c**). **b**, **c** *Z*-score normalized pseudocounts at the different time points after γ-irradiation of the 151 grouped (*Cl1–Cl8*) protein-coding DEGs. The *z*-score normalized pseudocounts are shown as a color gradient from blue (−2) over white (0) to red (2).

A pairwise Pearson correlation between the samples was calculated to analyze the differences within the replicates (measured for the 0, 1, and 4 h time points) and between the time points (Supplementary Table 2). Overall a strong correlation (>0.82) for all pairwise comparisons was observed. For one control replicate a 35% lower sequencing depth was observed, but the profile still correlated strongly with that of the second 0 h replicate and all other time points (0.82–0.94). Differential expression analysis of all samples (1, 2.5, 4, and 6 h after γ-irradiation), in comparison to the control (0 h; Supplementary Table 3, Supplementary Table 4), revealed 167 differentially expressed protein-coding genes (DEGs, Variance calculation per gene in DESeq2 over all samples; Supplementary Table 5). To further analyze the expression behavior of these DEGs we grouped them into clusters according to their expression at specific time points (Fig. 2A). Eight clusters (Fig. 2A; Cl1–8) were selected representing 90% of the 167 protein-coding DEGs that were further characterized as up- or downregulated (Fig. 2B, C; Cl1–8up/down; Supplementary Table 5). Among up-regulated genes, Cl1u–Cl4u contain genes that show enhanced expression at only one specific time point (Cl1u: 1 h; Cl2u: 2.5hu; Cl3u: 4 h; Cl4u: 6 h). Cl5–Cl8 show a constant chronological expression starting at 1 h (Cl8u), 2.5 h (Cl7u), or 4 h (Cl5u). Only DEGs in Cl6u show a more complicated expression profile as they were expressed at 2.5 h and 6 h but not 4 h leading to a possible oscillating behavior. Further analysis showed that DEGs in Cl5 and Cl6 were all upregulated (Fig. 2C), whereas most of the DEGs in Cl8 were downregulated.

**Fig. 3:**
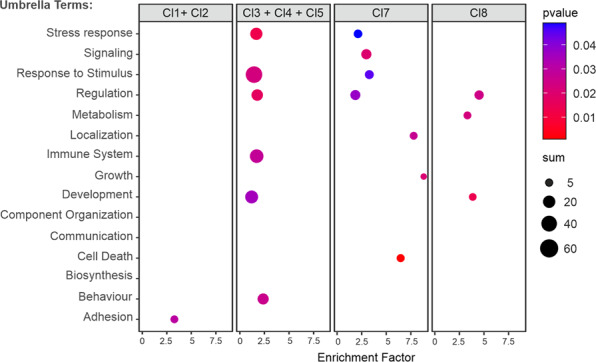
Assignment of DEGs to GO-terms based on statistical overrepresentation test and grouped umbrella terms. Shown are early response DEGs (Cl1 and Cl2), late response DEGs (Cl3–Cl5) as well as Cl7 and Cl8. Visualized are the enrichment factor (*x*-axis), the number of assigned DEGs (circle size) and adjusted p-value (color gradient 0.05 blue to 0.00 red).

To identify the cellular consequences related to these gene expression changes we combined the hierarchy “level 3” GO-terms of biological processes (Pantherdb; Gene Ontology released 2021-02-01, DOI: 10.5281/zenodo.4495804) to 20 umbrella terms (Supplementary Table 6) and performed an overrepresentation analysis of the DEGs within the specific clusters. For the early response genes (Cl1 and Cl2) only “adhesion” is an overrepresented umbrella term, whereas for the late response genes (Cl3–Cl5) six umbrella terms are overrepresented (Fig. 3). Most of the DEGs in constant chronological expression clusters (Cl7 and Cl8) and in late response clusters (Cl3, Cl4, and Cl5) are involved in “regulation” of cellular processes (Fig. 3). The processes of “stress response” and “response to stimulus” are overrepresented in Cl7, while “development” is overrepresented in Cl8. The process of “cell death” was only observed in clusters showing expression after 2.5 h which correlates well with the activation kinetics of TAp63α (Fig. 1).

**Fig. 4:**
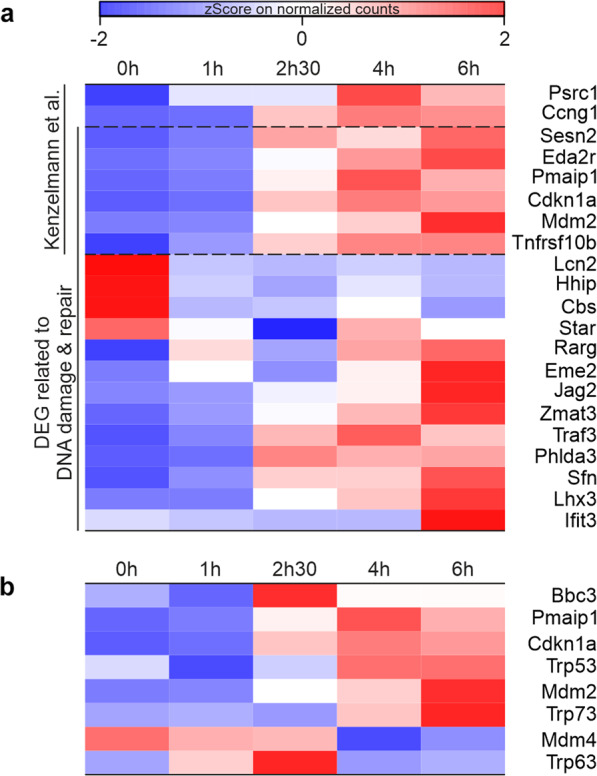
DEGs from our study related to GO-terms including “Apoptosis”, “Necroptosis”, and/or “DNA damage and repair”. **a** The six DEGs in this category and the two additional DEGs involved in cell cycle arrest, which are all known from Kenzelmann et al. to be regulated by p53, are indicated [23]. *Z*-score normalized pseudocounts are represented between −2 (blue) and 2 (red). **b** Expression profiles of members of the p53 family, *Mdm4*, and *Bbc3* (PUMA), which do not reach statistical significance in our study but are important for regulating apoptosis. For comparison, expression data for the DEGs *Pmaip1*, *Cdkn1a*, and *Mdm2* are shown.

To focus on important processes triggered by γ-irradiation we used a refined word mining approach selecting for GO-terms including “DNA-damage and repair”, “Necrosis” and/or “Apoptosis”. Only 19 out of the 151 DEGs (Fig. 4) could be directly assigned to at least one of these processes. A previous study from Kenzelmann et al. [23] identified 64 genes in mouse embryo fibroblasts that are directly regulated by p53 6 h after treatment with doxorubicin (Supplementary Table 7). Of these 64 genes, we found six in our list of DEGs assigned to “Apoptosis” (Fig. 4) and two involved in cell cycle arrest (Psrc2 and Ccng1). Only four genes had a ~1.6-fold higher normalized expression before γ-irradiation than after the treatment (Lcn2, Hhip, Star, and Cbs). As these four genes are also involved in metabolic processes and development, they seem less important for the acute stress response following γ-irradiation. From the nine other DEGs, six showed an average 1.33-fold higher normalized expression after 2.5 h (Jag2, Zmat3, Traf3, Phlda3, Sfn, and Lhx3; Supplementary Table 3). These results lead to the conclusion that a major transcriptional change occurs after 2.5 h following γ-irradiation, which activates genes involved in DNA damage response and apoptosis. As TAp63α becomes fully transcriptionally active at 2.5 h, it seems likely that the switch in expression program at 2.5 h is mainly due to the activation of p63.

The main effectors of p63-based induction of apoptosis in oocytes are the two BH3 only proteins PUMA (*BBC3*) and NOXA (*Pmaip1*) [21]. Only *Pmaip1* could be identified as a DEG after 2.5 h assigned in cluster Cl7. Although *BBC3* is not detected as one of the DEGs (adj. *p*-value > 0.05), *BBC3* gets upregulated at 2.5 h but to a lesser extent at 4 and 6 h. We also investigated the expression of *Trp63*, *Trp53*, and *Trp73* as well as *Mdm4*. Of these, only *Trp73* shows an increasing upregulation over the investigated period. In contrast, *Mdm4* becomes downregulated following irradiation (Fig. 4b; Supplementary Table 3) which is in stark contrast to the p53-focused study in MEFs [23] where *Mdm4* is as strongly upregulated as *Mdm2*.

To validate the RNA-seq results for some selected genes we used qPCR of samples taken 2.5 h and 6 h after γ-irradiation and compared the induction levels relative to the sample before irradiation. The results confirmed the upregulation of *Cdkn1a*, *Pmaip1*, *Bbc3*, *Mdm2*, *Ccgn1*, *TP73*, and *Eda2r* and showed that *TP53* and *Mdm4* do not get upregulated (Supplementary Fig. 2).

### Comparison with other p63 regulated transcriptome analyses

p63 is not only expressed in oocytes, it is also abundant in the basal compartment of stratified and pseudo-stratified epithelial tissues [24, 25]. These cells, however, express a different isoform, ΔNp63α, which lacks the N-terminal 69 amino acids [24] including the transactivation domain localized between N8 and E24 [26]. This isoform is essential for the proliferation and differentiation of keratinocytes [27, 28], the role of ΔNp63α in keratinocytes is, however, more complex than the role of TAp63α in oocytes. ChIPseq experiments have identified many thousand binding sites, many of them in enhancer regions [29–31]. ΔNp63α is inactive on classical p53 target promotors (such as *Cdkn1a* or *Mdm2*) [24] and rather acts as a suppressor of those genes. Certain genes important for keratinocyte development are, however, regulated by ΔNp63α [32]. Several different RNA-seq and ChIPseq analyses have been performed in different cell types [33, 34]. A recent study has used induced pluripotent stem cells (iPSCs) derived from human dermal fibroblasts of healthy individuals and patients expressing the R204W or R304W mutation in the p63 DBD. These mutations abolish DNA binding and cause the EEC syndrome [35–37]. These iPSCs were induced to differentiate into keratinocytes [31] and RNA-seq analysis resulted in the identification of 296 upregulated genes in ΔNp63α mutated cells (potential loss of suppressor function) and 241 genes that were downregulated. Comparison with our data showed that the list of downregulated genes contains three of our DEGs while the list of upregulated genes contains eight DEGs from our TAp63α/oocyte study (Supplementary Table 8). Two of these genes (*EDA2R* and *PMAIP1*) are involved in apoptosis and their upregulation by loss of ΔNp63α or by activation of TAp63α suggests that ΔNp63α acts as a repressor of these genes in keratinocytes.

In addition, we compared our DEG list with a recently published list compiled from all so far published p63 RNA-seq/ChIPseq studies that contain 180 targets [38] (138 upregulated, 42 downregulated genes). This comparison yielded a small number of overlapping genes (Supplementary Table 7), demonstrating again that both isoforms have very different and almost non-overlapping functions.

### Interferon-stimulated genes are upregulated at the 6 h time point

**Fig. 5:**
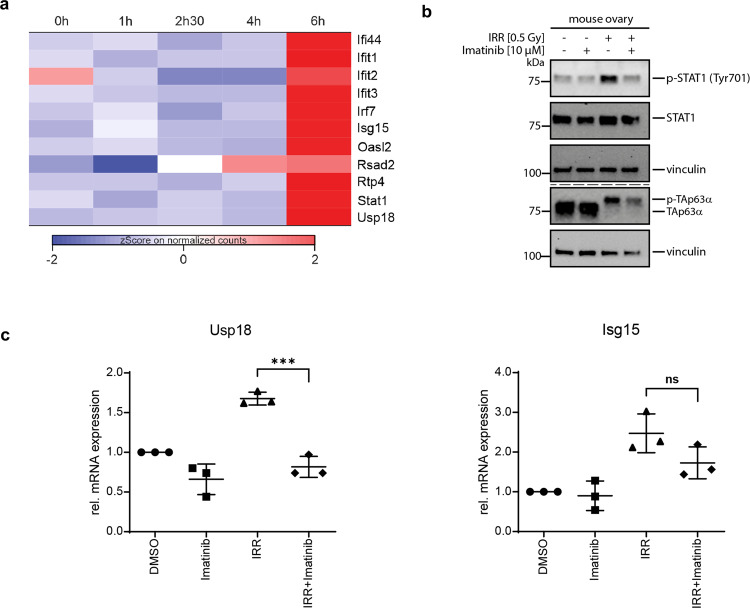
Expression kinetics of interferon-induced genes based on z-score normalized pseudocounts. **a** Several genes show a specific upregulation at the 6 h time point. Only Ifit1, Ifit3, Isg15, and Usp18 reach statistical significance in our analysis (*p*-value below 0.05 for 6 h). **b** The level of STAT1 and phosphorylated STAT1 were compared at the 6 h time point. Irradiation results in robust STAT1 phosphorylation, which is strongly reduced by adding imatinib before irradiation. The phosphorylation status of TAp63α was probed as well, showing that irradiation results in phosphorylation, which is not prevented by treatment with imatinib. **c** Usp18 and Isg15 mRNA levels were determined by real-time PCR 6 h after γ-irradiation of mouse ovaries in the presence or absence of imatinib. Irradiation (IRR) results in upregulation of both genes, which is inhibited by treatment with imatinib. The mRNA levels of non-irradiated ovaries treated with DMSO served as reference and were set to 1. The foldchange of the mRNA levels of the other conditions relative to the DMSO control was determined (*n* = 3).

Our DEG list also contains three interferon-stimulated genes, *Ifit1*, *Ifit3*, and *Isg15* as well as the Isg15 specific Dub *Usp18*. All four genes show a very steep expression increase at 6 h (Fig. 5a). Based on this observation we re-investigated the expression behavior of other interferon-stimulated genes. Indeed many of them show a similar expression pattern (Fig. 5a). To investigate if these genes are potentially regulated by p63, we analyzed their promotor regions which showed that a small number of these genes including Ifit1/2/3 and Rasd2 have a weak p63 binding motif (Supplementary Table 9). The time difference between transcription of apoptosis-involved genes (upregulated at 2.5 h) and this group of interferon-regulated genes (sharply upregulated at 6 h) combined with the lack of strong p63 binding sites triggered our search for alternative mechanisms. Typically, interferon-stimulated genes are upregulated following activation of cytokine receptors [39]. Ligand-induced dimerization activates JAK kinases which further results in phosphorylation of STAT transcription factors [40]. An alternative route is the activation of STAT proteins via the soluble tyrosine kinase c-Abl. c-Abl itself gets activated via ATM following the detection of DNA damage [41, 42] which is also the start point of the ATM-CHK2-CK1-TAp63α signal cascade. Interestingly, the effects of c-Abl inhibitors on the survival of oocytes treated with chemotherapeutics have been reported [16, 43, 44]. In order to investigate the potential role of c-Abl in the regulation of interferon-stimulated genes, we first analyzed the phosphorylation state of STAT1. Irradiation of mouse ovaries with 0.5 Gy resulted in strong STAT1 phosphorylation at the 6 h time point (Fig. 5b) which was inhibited by the treatment with the c-Abl inhibitor imatinib. This suggests that STAT1 can be activated intracellularly by c-Abl. In qPCR experiments, we further demonstrated that treatment with imatinib reduced mRNA levels of *Isg15* and *Usp18* in ovaries analyzed 6 h after irradiation, further supporting an intracellular pathway (Fig. 5c).

### Changes in expression following lower irradiation dosage

**Fig. 6:**
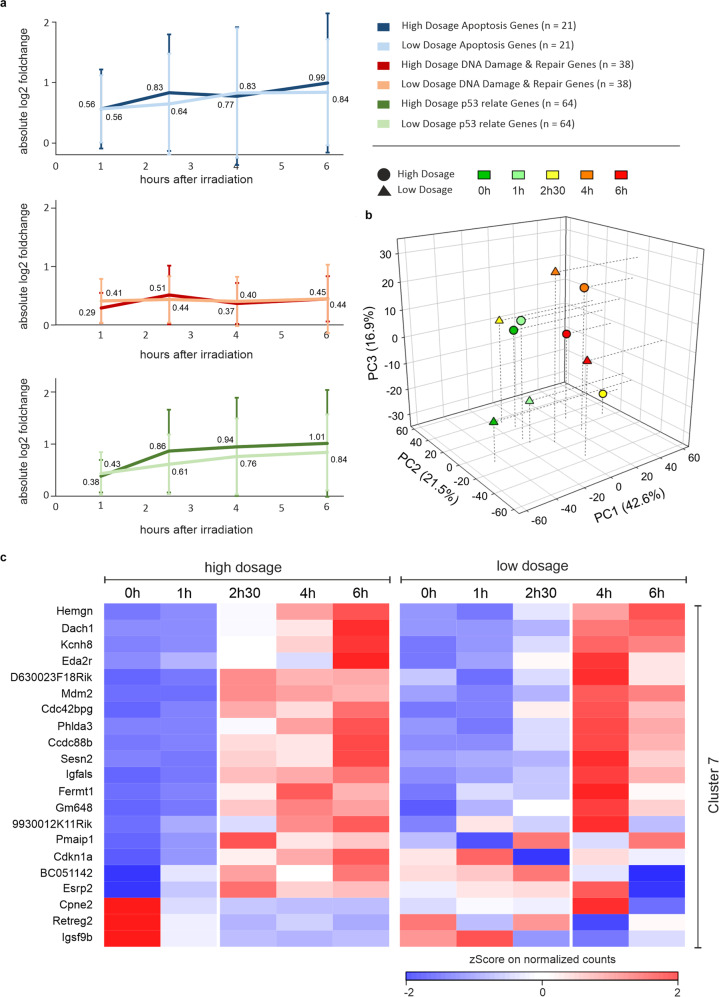
Comparison of transcriptional changes following irradiation with low and high dosage. **a** Analysis of genes at high and at low dosage assigned to “Apoptosis”, “DNA damage and repair” (Go-Term word mining), or p53-regulated genes based on Kenzelmann et al. [23]. **b** Principal component analysis of the first three components (PC1–PC3). Low dosage (triangle) and high dosage (circles) samples of different time points are colored (dark green (0 h), green (1 h), yellow (2.5 h), orange (4 h), red (6 h)). **c** Comparison of the expression kinetics of DEGs from Cl7 between high and low dosage samples based on *z*-score normalized pseudocounts.

Next, we investigated whether a slower induction of apoptosis induced by a lower irradiation dosage of 0.2 Gy allows oocytes to initiate a DNA repair response (Supplementary Table 10). This dosage is still lethal for the majority of the primary oocytes, however, oocyte death occurs slower [7]. We focused only on genes assigned to biological processes “DNA-damage and repair” and/or “Apoptosis” as well as p53 regulated genes based on Kenzelmann et al. [23] (Fig. 6a, Supplementary Table 11). The RNA transcript level of genes involved in apoptosis increased over time at high and low dosage, however, the fold-change at low dosage remained lower (Fig. 6a). Similar behavior is seen for the p53 target genes (this group has some overlap with the apoptosis group, Supplementary Table 11). In contrast, nearly no increase in gene transcripts related to DNA repair was detected, neither at low nor at high dosage (Fig. 6a, Supplementary Table 11). To investigate a correlation between the expression changes and the slower oocyte death we performed a principal component analysis (PCA; Fig. 6b; Supplementary Table 12). The PCA showed that ~80% of the variance within the samples can be explained by the first three principal components (PCs). Most of the time point samples of the different γ-irradiation dosages (Fig. 6b) are relatively close to each other in the first three PCs except for the 2.5 h time points. Furthermore, the samples of 0 and 1 h can be clearly separated from the 4 and 6 h time points. Only for the 2.5 h time points, a separation is visible between high and low dosage. The low dosage sample at 2.5 h is closely located relative to the 0 and 1 h clusters, whereas the high dosage sample at 2.5 h is more closely related to the 4 and 6 h clusters. This suggests that changes in gene expression are delayed in the low dosage samples, further supported by the expression profiles of Cl7 (Fig. 6c) where low dosage treatment results in a delayed response (some genes like *Cdkna1* show a more complex behavior, which is most likely due to experimental errors based on the fact that no replicates were obtained. The goal of the low dosage study, however, was not to investigate individual genes but entire clusters relative to the high dosage data).

**Fig. 7:**
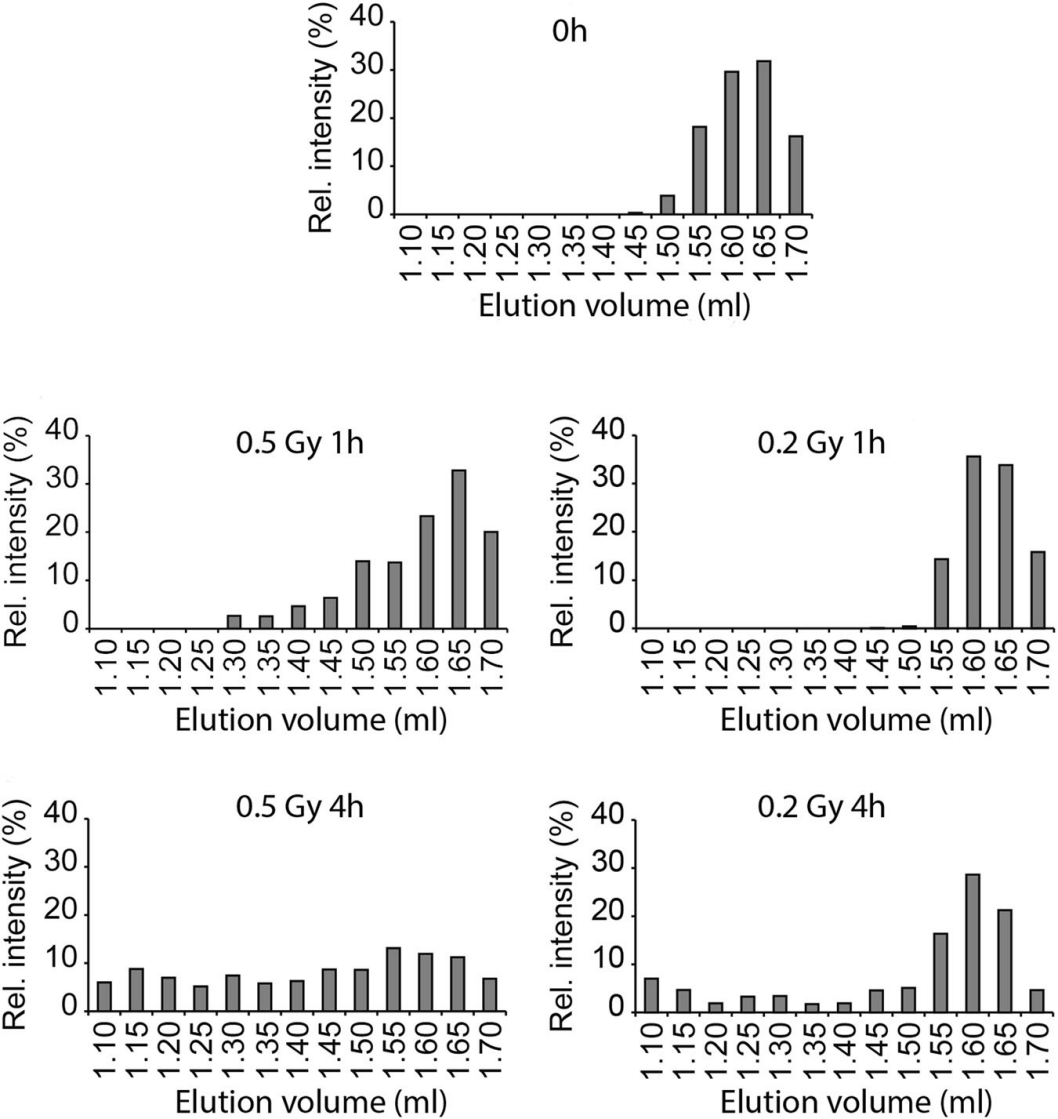
Comparison of the size exclusion profiles of TAp63α from mouse ovary extracts irradiated with either 0.2 Gy, 0.5 Gy, or nonirradiated (0 h). Each fraction corresponding to an elution volume of 50 µl was probed for the presence of TAp63α by Western blot analysis (Supplementary Fig. 3). Quantification using bar graphs representing the percentage of a certain fraction of the total TAp63α signal is shown. Dimeric TAp63α elutes at approx. 1.60 mL elution volume, tetrameric TAp63α elutes at approx. 1.3 mL, shows, however, due to the open conformation a wider distribution.

To correlate this activation delay with the TAp63α activation kinetics we performed size exclusion chromatography analysis of extracts prepared from irradiated mouse ovaries at the different times points (Fig. 7). These data show that at the 0.2 Gy 4 h time point, TAp63α tetramerization just started, similar to the 1 h time point following irradiation with 0.5 Gy. These data confirm that there is a delayed activation of TAp63α, consistent with a delayed transcription of apoptosis genes.

## Discussion

The presence of TAp63α in resting oocytes [45] makes them particularly sensitive to DNA damage. Oocyte death is directly linked to the activation of TAp63α which results in the expression of PUMA and NOXA [21]. Other classical p53-target genes also get transcribed, for example, *Cdkn1a* and *Mdm2*. The reason for *Cdkn1a* expression in oocytes, which are arrested in the cell cycle, is not immediately evident. Other genes involved in cell cycle arrest, *Psrc2* and *Ccng1*, get expressed as well. In addition, Phlda3, an inhibitor of Akt signaling, is also expressed. Akt plays a major role in the activation of dormant primordial follicles [46–50]. Inhibiting Akt also prevents inactivation of the cell cycle inhibitor p27 [51, 52] and TSC2, an inhibitor of mTOR [53]. In mice, lack of TSC2 results in activation of primordial follicles [54]. Similarly, Sestrin 2, an inhibitor of the TORC1 signaling pathway gets expressed. These data show that part of the cellular response to γ-irradiation is a strong inhibition of re-entry into the meiotic process. Our results are consistent with a recent study that used single-cell RNA-sequencing of primordial follicle oocytes 12 h after cyclophosphamide injection in a human ovarian xenograft model. This study showed that the depletion of the ovarian reserve is not due to the “burn-out” mechanism as no activation of the PI3K/PTEN/Akt pathway could be detected [55].

In contrast to the apoptotic program, we could not detect the upregulation of a DNA repair program. In contrast to bacteria, which have a strong induction of DNA repair genes after sensing DNA damage (SOS response), only a few genes are upregulated after DNA damage in mammalian cells [56]. The mammalian DNA damage response rather requires posttranslational modifications and re-localization than enhanced transcription. These results indicate that the decision of life and death is based on the strength of the apoptotic signal rather than the relative expression of apoptotic and DNA repair pathways. However, if oocytes survive they repair DNA efficiently [13, 21], mainly through the homologous recombination pathway [57, 58].

Oocytes are known to critically depend on contacts to the surrounding granulosa cells and changes in adhesion will have a strong impact on their survival. Changes in the expression of genes involved in adhesion are among the earliest detected and are mostly downregulated. Other genes that are downregulated already at the 1 h time point are involved in developmental processes and metabolic functions (Cl8). This suggests that developmental processes are stopped and changes in the metabolism occur as soon as cellular damage is detected. For all genes showing an early response, it is unlikely that p63 is involved.

One surprising result of this study is that at the 6 h time point interferon-stimulated genes are upregulated when cells have fully activated apoptosis. The upregulation of *Isg15* (and other interferon-stimulated genes) has been linked not only to viral infection but also to DNA damage [59, 60]. While some interferon-stimulated genes have p63 binding sites, a more likely pathway is via STAT transcription factors. These can be activated not only through cytokine receptors [39] but also intracellularly by c-Abl [61]. Interestingly, the effects of imatinib on the survival of oocytes following the treatment with cisplatin have been reported but also controversially discussed [43, 62]. While it has been shown by gene knockout studies as well as inhibitor studies that c-Abl is not involved in the activation of dimeric TAp63α to its tetrameric state [16, 44], it is possible that c-Abl phosphorylates TAp63α further. For TAp73α, which is constitutively tetrameric [63] and therefore regulated by different mechanisms, interaction with and phosphorylation by c-Abl have been reported [64–66]. Alternatively, the imatinib effects observed in oocytes could be related to the DNA damage triggered activation of STAT proteins via c-Abl and the concomitant upregulation of interferon-stimulated genes. Although imatinib inhibits not only c-Abl but also the receptor tyrosine kinases c-Kit and PDGF-receptor, c-Abl is most likely the critical kinase due to its activation by DNA damage. Thus, our data suggest that rather c-Abl activated STAT proteins and not p63 are responsible for the observed effect. The reason for the upregulation of interferon-stimulated genes is currently not known but could be important to orchestrate phagocytosis of the dying oocyte as Isg15 also gets secreted and acts as a chemoattractant for example for neutrophils [67].

## Material and methods

### Oocyte RNA isolation

Animal care and handling were performed according to the World Health Organization (Geneva, Switzerland) guidelines. Five-day-old (P5) female CD-1 mice were purchased from Charles River Laboratories. Ovaries were harvested, transferred to sterile 96-well plates with 50 µl α-MEM (+l-Glu, Gibco) supplemented with 10% fetal bovine serum (Gibco), 1× penicillin/streptomycin (Gibco), 0.2 mg/ml Na-pyruvate (Gibco), 2 mg/ml *N*-acetyl-l-cysteine (Sigma) and ITS liquid media supplement (100×) (Sigma) cultured at 37 °C with 5% CO_2_ overnight [68]. The “Tierschutzbeauftragte” approved the protocol for harvesting mouse ovaries of the Goethe University Frankfurt/Main. Ovaries were irradiated with 0.5 or 0.2 Gy; (Caesium137 was the source of irradiation. The dose rate was 109 rad/min). The ovaries were transferred at time points 0, 1, 2.5, 4, and 6 h after the irradiation into 40 µl Trypsin (0.25%) solution. To dissolve the ovary a yellow tip pipette was used and the solution was pipetted for 5 min. When the solution turned cloudy the ovaries were solubilized. Pipetting was continued for another minute to separate the oocytes from the follicular cells. This solution was centrifuged for 45 s at 1000 rpm in a table centrifuge to separate the follicular cells from the oocytes. This washing step was repeated until the oocytes were clean. After each centrifugation step, the upper layer (follicular cells) was transferred to a new tube. Once the oocytes were clean the last centrifugation step (5 min at 3000 rpm) followed to pellet the oocytes and discard the supernatant. These oocytes were resuspended in 30 µl PicoPure Extraction Buffer and placed for 30 min at 42 °C before freezing the samples. The Samples were kept at −80 °C until the RNA extraction was continued as described in the Pico Pure Kit protocol. The resulting RNA was reverse transcribed by using the SMARTER kit (Clontech), according to the manufacturer’s protocol. For each time point, four ovaries were used.

### Differential expression analysis

For differential expression analysis, RNA-seq experiments were used containing five different points of time (0, 1, 2.5, 4, and 6 h) after treatment with either 0.5 or 0.2 Gy. The 30 bp paired-end reads of RNA-seq samples on illumina HiSeq were produced as duplicates for points of time (0, 1, and 4 h) and once for 2.5 and 6 h. All RNA-seq datasets were mapped on the reference genome of the mouse (Ensembl version 92) via salmon (v 0.9.1). The expression estimation algorithm of salmon precisely estimates pseudocounts instead of read counts and allows a differentiation on a transcript- as well as gene-level. For differential expression analysis all alternative splice variants of the single transcripts were assigned to the annotated gene (mouse genome version GRCm38). After pairwise Pearson correlation of all datasets normalization and differential expression was performed via DESeq2 for high dosage samples. As not all samples had at least two replicates we used the implemented estimateDispersion function of DESeq2 to estimate from the complex design over all samples with and without replicates the per gene expression via Cox Reid-adjusted profile likelihood. For this size factor of the libraries was estimated as well as a Wald-test (default parameters and adding beta_prior = True) was performed. All genes differentially expressed (DE) in at least one condition compared to 0 h (log2 fold change > 1; adjusted *p*-value < 0.05) were called as DE gene. For multiple-testing correction, we used the (Benjamini–Hochberg) parameter for the *p*.adjust function.

The RNAseq data are deposited in the GEO data bank with the accession code GSE184704.

### Functional annotation and literature set comparison of DE genes

For functional annotation of the genes, Gene Ontology (GO) terms were assigned to the single genes via G-Profiler (source). G-Profiler annotates for each gene all GO-terms corresponding to the functional hierarchy depth within the GO-graph. GO-terms related to a “biological process” (hierarchy level 1) have been used as root. In the GO-graph for the mouse genome 29 level2 GO-terms, 422 level3 GO-terms, and 2121 level4 GO-terms occur (e.g., “signaling” level2; “cytokine production” level3; “type I interferon production” level4). For unique functional annotation of the DE genes hierarchy “level 3” was used in this study to create word clouds. For the representation of the overall functions within a DE, cluster word clouds were created, at which the abundance of a “level 3” GO-term throughout the expression cluster is represented by the font size (font size 10 for the lowest abundance; font size 30 for the highest abundance per cluster). Level 3 GO-terms that represent a similar function were combined with umbrella terms to preserve a clear overview (Supplementary Table 6). To calculate the abundance of a GO-term each Gene with this GO-term annotated is counted once. A single gene can therefore be counted for several GO-terms, which means the sum of all GO-abundances do not add up to the number of genes per cluster. Each gene with a corresponding GO term was counted once per umbrella GO term. To compare genes that were observed to be DE to known literature data, all 64 p53 related genes proposed by Kenzelmann Broz et al. [23], were compared to the 167 DE protein-coding genes. GO-Terms including the terms “DNA-damage/ -repair”, “Necroptosis”, or “Apoptosis” were extracted from GO-hierarchy and assigned to umbrella terms.

### Size exclusion chromatography analysis and western blotting of TAp63α from ovarian extracts

Ovaries were harvested from P5 mice and were either irradiated with 0.5 Gy, 0.2 Gy, or non-irradiated as described above. For each size exclusion chromatography experiment 16 ovaries were lysed by mechanical force in 50 mM sodium phosphate, pH = 7.2, 150 mM NaCl, 0.1% Triton X-100, EDTA free protease inhibitor cocktail (Roche), and phosphatase inhibitor cocktail (Roche) in a total volume of 70 µl. After centrifugation at 20,000×*g* for 15 min at 4 °C the supernatant was injected in a Superose 6 PC 3.2/30 column (GE Healthcare) equilibrated with 50 mM sodium phosphate, 100 mM NaCl, EDTA free protease inhibitor cocktail, and phosphatase inhibitor cocktail at 4 °C and eluted as described above. Collected fractions were separated using 10% Bis–Tris NuPAGE gels (Invitrogen) in MOPS buffer at 4 °C and subsequently transferred on a Hybond-P membrane (GE Healthcare) using an XCell II blot module (Invitrogen). Blots were then blocked with 5% skim milk in TBS buffer containing 0.1% Tween-20 and probed overnight at 4 °C with 4A4 antibody (gift from Frank McKeon) [7]. Detection was performed using goat anti-mouse IgG-Fab-HPR (A9917, Sigma Aldrich). Blots were quantified using Biometra BioDocAnalyze 2.0 software.

For c-Abl inhibition experiments, four ovaries per condition from P8 mice were treated with 10 µM imatinib (SML1027, Sigma) for 1 h and subsequently irradiated with 0.5 Gy. Ovaries were collected in 10 µl lysis buffer A (50 mM Tris pH 8.0, 100 mM NaCl, 0.5 mM TCEP, 2 mM MgCl_2_, 1× PhosSTOP, 1× cOmplete) and lysed with a pestle and multiple freeze–thaw cycles. Afterward, 10 µl lysis buffer B (lysis buffer A + 40 mM CHAPS) and 1 µl Benzonase (Merck) was added to the samples and incubated on ice for 1 h. The supernatant was separated on 4-12% SDS-PAGE Mini-PROTEAN TGX gels (Bio-Rad) and blotted using the semidry Trans-Blot Turbo Transfer System (Bio-Rad). Blots were further processed as described before. Anti-p63-α (D2K8K XP, Cell Signaling), anti-Phospho-STAT1 (Tyr701) (58D6, Cell Signaling), anti-STAT1 (D1K9Y, Cell Signaling), and anti-Vinculin (7F9, Santa Cruz) were used for protein detection. As secondary antibody a goat anti-rabbit IgG (H + L) (AB_2307391, Jackson ImmunoResearch) was used.

### Kinetic measurements

The concentrations of TAp63α, Msy2, cleaved PARP1 and Pro-caspase9 were analyzed by SDS-PAGE and Western Blotting at different time points between 1 and 45 h following irradiation with 0.5 Gy as well as before irradiation. For each time point, one ovary was harvested and irradiated as described above. Following irradiation 10 µL SDS loading buffer was added per ovary, the sample was heated for 10 min to 95 °C and subsequently centrifuged at 3000 rpm for 2 min. Five microlitres of each sample were mixed with 5 µL of 2× Laemmli sample buffer and loaded on a 4–12% Bis–Tris PAGE gel. Gels were run at 4 °C at a constant voltage of 200 V. Western blot analysis was performed using an XCell II blot module (Invitrogen) to transfer proteins to a polyvinylidene fluoride membrane. The membrane was blocked for 1 h with 5% skim milk and incubated overnight with the corresponding primary antibodies. The membrane was washed three times with TBS-T buffer (TBS buffer with 0.05% TWEEN) before incubation with the secondary HPR-conjugated antibody for 1 h. The membrane was washed three times with TBS-T buffer and treated with ECL Plus WB Detection Systems solution (GE Healthcare) for 5 min. Signals were detected with a Lumi Imager F1 documentation system.

For the detection, the following primary antibodies were used: H-129 (Santa Cruz Biotechnology) (anti-TAp63α), N-13 (Santa Cruz Biotechnology) (anti-Msy2), D214 (Cell Signaling Biotechnology) anti-cleaved PARP, C9 (Cell Signaling Biotechnology) anti-Caspase 9. As secondary antibodies (all from Sigma Aldrich) goat anti-rabbit IgG-HPR (DC03L), goat anti-mouse IgG-Fab-HPR (A9917), and rabbit anti-goat IgG-HRP (AP106P) were used.

### Real-time quantitative PCR

Real-time quantitative PCR was performed with three independent sets of samples. For each condition per set four dissected ovaries were pooled. Oocytes were isolated by trypsin-digestion and multiple centrifugation steps. Total RNA was extracted applying the PicoPure RNA Isolation Kit (Applied Biosystems) with on-column DNAseI (Qiagen) digestion and subsequently subjected to reverse transcription with random primers using the RETROscript Kit (Ambion) followed by cDNA amplification with the TaqMan PreAmp Kit (ThermoFisher Scientific). Real-time quantitative PCR to determine the fold-induction of p63 target genes was performed with TaqMan Gene Expression Assays (ThermoFisher Scientific) using a LightCycler 480 (Roche). For one biological set, each sample and TaqMan assay probe combination was measured in duplicates. All Kits were used according to the manufacturer’s instructions. Target gene signals were referenced to the housekeeping gene TBP and each biological replicate was normalized to its non-irradiated sample before calculating the mean fold induction and standard deviation. The statistical significance was determined by the ordinary one-way ANOVA using GraphPad Prism (Version 8.0.2.).

In experiments, in which RNA was isolated directly from ovaries, the AllPrep DNA/RNA/Protein Mini Kit (Cat. No. 80004, Qiagen) was employed for RNA isolation. cDNA was synthesized from the isolated RNA using the RevertAid First Strand cDNA Synthesis Kit (Cat. No. K1622, Thermo Scientific) and the supplied random hexamer primers. The real-time PCR reactions were performed on an iCycler (BioRad cxn96 or connect / Applied Biosystems Step One Plus). The reactions were carried out in biological triplicates with four ovaries per experiment using EvaGreen (Cat. No. 27490, Axon). For normalization and calculation of relative expression levels, the mRNA levels of the housekeeping gene GAPDH were used. The mRNA levels of the non-irradiated but DMSO treated ovaries were used as a reference and set to 1. The foldchange of the mRNA levels of all other conditions was determined relative to the DMSO control (*n* = 3). Significance was evaluated by *t*-test in GraphPad Prism, ***P < 0.001.

Real-time PCR primers (TaqMan):

**Table:** 

Target	ID	Cat.number
Bbc3	Mm00519268_m1	4453320
Pmaip1	Mm00451763_m1	4453320
BAX	Mm00432051_m1	4453320
Cdkn1a	Mm04205640_g1	4448892
Mdm2	Mm01233136_m1	4453320
Mdm4	Mm00484944_m1	4448892
Ccng1	Mm00438084_m1	4448892
Lrdd	Mm00502625_g1	4448892
Eda2R	Mm00723601_m1	4448892
p53	Mm01731290_g1	4448892
p73	Mm01263636_m1	4448892
Ybx2	Mm01250826_gH	4448892
Tbp	Mm00446971_m1	4453320
	TaqMan® Gene Expression Master Mix	4369016
	TaqMan® PreAmp Master Mix Kit	4384267

Real-time PCR primers (EvaGreen):

GAPDH: fw: 5′-gtttctataaattgagcccgc-3′/rev: 5′-tgtaaaccatgtagttgaggt-3′

Usp18: fw: 5′-atgactcacatgtttgttgg-3′/rev: 5′-cttcgtgtaaaccaagagatag-3′

Isg15: fw: 5′-atggaggacttttgggatag-3′/rev: 5′-agaggcagagctttttattg-3′

### P63 binding motif analysis at promoter regions

TSS regions were identified from the mouse genome mm10 Ensembl annotation using genomepy (doi: 10.21105/joss.00320). Promoter regions were defined as 2 kb up- and downstream of the TSS regions of genes of interest. The TP63 motifs “GM.5.0.p53.0001” and “GM.5.0.p53.0004” were then selected from the gimme motifs database gimme.vertebra.v5.0 and used for detection of the presence of TP63 motifs with multiple rounds of gimme scan (doi: 10.1101/474403), with a minimum motif similarity of 0.85, in the promoter regions.

## Supplementary information

Supplementary Figures 1-3

Suppl_Table_1_Sequencing_Information

Suppl_Table_2_Pearson_correlation

Suppl_Table_3_DESeq2

Suppl_Table_4_FDR

Suppl_Table_5_Cluster_genes

Suppl_Table_6_GO-to-Umbrella

Suppl_Table_7_otherStudies

Suppl_Table_8_ipsc

Suppl_Table_9_TP63motif_promoters_0.85

Suppl_Table_10_Sample_low_dosage

Suppl_Table_11_RNAseq_samples_lowhigh

Suppl_Table_12_Foldchanges_High_Low

aj-checklist

## Data Availability

All data needed to evaluate the conclusions in the paper are present in the article and its supplementary material. Additional data related to this paper may be requested from the corresponding authors.

## References

[1] Gebel J, Tuppi M, Krauskopf K, Coutandin D, Pitzius S, Kehrloesser S (2017). Control mechanisms in germ cells mediated by p53 family proteins. J Cell Sci.

[2] Lena AM, Rossi V, Osterburg S, Smirnov A, Osterburg C, Tuppi M (2021). The p63 C-terminus is essential for murine oocyte integrity. Nat Commun.

[3] Candi E, Melino G, Toth A, Dotsch V (2021). Mechanisms of quality control differ in male and female germ cells. Cell Death Differ.

[4] Hunt PA, Hassold TJ (2002). Sex matters in meiosis. Science.

[5] Levine AJ, Tomasini R, McKeon FD, Mak TW, Melino G (2011). The p53 family: guardians of maternal reproduction. Nat Rev.

[6] Gebel J, Tuppi M, Sanger N, Schumacher B, Dotsch V. DNA damaged induced cell death in oocytes. Molecules. 2020;25:5714–38. 10.3390/molecules25235714 PMC7730327 33287328

[7] Suh EK, Yang A, Kettenbach A, Bamberger C, Michaelis AH, Zhu Z (2006). p63 protects the female germ line during meiotic arrest. Nature.

[8] Livera G, Petre-Lazar B, Guerquin MJ, Trautmann E, Coffigny H, Habert R (2008). p63 null mutation protects mouse oocytes from radio-induced apoptosis. Reproduction.

[9] Derry WB, Putzke AP, Rothman JH (2001). Caenorhabditis elegans p53: role in apoptosis, meiosis, and stress resistance. Science.

[10] Schumacher B, Hofmann K, Boulton S, Gartner A (2001). The C. elegans homolog of the p53 tumor suppressor is required for DNA damage-induced apoptosis. Curr Biol.

[11] Ou HD, Lohr F, Vogel V, Mantele W, Dotsch V (2007). Structural evolution of C-terminal domains in the p53 family. EMBO J.

[12] Deutsch GB, Zielonka EM, Coutandin D, Weber TA, Schafer B, Hannewald J (2011). DNA damage in oocytes induces a switch of the quality control factor TAp63alpha from dimer to tetramer. Cell.

[13] Bolcun-Filas E, Rinaldi VD, White ME, Schimenti JC (2014). Reversal of female infertility by Chk2 ablation reveals the oocyte DNA damage checkpoint pathway. Science.

[14] Serber Z, Lai HC, Yang A, Ou HD, Sigal MS, Kelly AE (2002). A C-terminal inhibitory domain controls the activity of p63 by an intramolecular mechanism. Mol Cell Biol.

[15] Straub WE, Weber TA, Schafer B, Candi E, Durst F, Ou HD (2010). The C-terminus of p63 contains multiple regulatory elements with different functions. Cell Death Dis.

[16] Tuppi M, Kehrloesser S, Coutandin DW, Rossi V, Luh LM, Strubel A (2018). Oocyte DNA damage quality control requires consecutive interplay of CHK2 and CK1 to activate p63. Nat Struct Mol Biol.

[17] Cesaro L, Pinna LA (2015). The generation of phosphoserine stretches in phosphoproteins: mechanism and significance. Mol Biosyst.

[18] Knippschild U, Kruger M, Richter J, Xu P, Garcia-Reyes B, Peifer C (2014). The CK1 family: contribution to cellular stress response and its role in carcinogenesis. Front Oncol.

[19] Coutandin D, Osterburg C, Srivastav RK, Sumyk M, Kehrloesser S, Gebel J, et al. Quality control in oocytes by p63 is based on a spring-loaded activation mechanism on the molecular and cellular level. Elife. 2016;5:e13909. 10.7554/eLife.13909 PMC4876613 27021569

[20] Gebel J, Tuppi M, Chaikuad A, Hotte K, Schroder M, Schulz L (2020). p63 uses a switch-like mechanism to set the threshold for induction of apoptosis. Nat Chem Biol.

[21] Kerr JB, Hutt KJ, Michalak EM, Cook M, Vandenberg CJ, Liew SH (2012). DNA damage-induced primordial follicle oocyte apoptosis and loss of fertility require TAp63-mediated induction of Puma and Noxa. Mol Cell.

[22] Zerbino DR, Achuthan P, Akanni W, Amode MR, Barrell D, Bhai J (2018). Ensembl 2018. Nucleic Acids Res.

[23] Kenzelmann Broz D, Spano Mello S, Bieging KT, Jiang D, Dusek RL, Brady CA (2013). Global genomic profiling reveals an extensive p53-regulated autophagy program contributing to key p53 responses. Genes Dev.

[24] Yang A, Kaghad M, Wang Y, Gillett E, Fleming MD, Dotsch V (1998). p63, a p53 homolog at 3q27-29, encodes multiple products with transactivating, death-inducing, and dominant-negative activities. Mol Cell.

[25] Marshall CB, Mays DJ, Beeler JS, Rosenbluth JM, Boyd KL, Santos Guasch GL (2016). p73 is required for multiciliogenesis and regulates the Foxj1-associated gene network. Cell Rep.

[26] Krauskopf K, Gebel J, Kazemi S, Tuppi M, Lohr F, Schafer B (2018). Regulation of the activity in the p53 family depends on the organization of the transactivation domain. Structure.

[27] Yang A, Schweitzer R, Sun D, Kaghad M, Walker N, Bronson RT (1999). p63 is essential for regenerative proliferation in limb, craniofacial and epithelial development. Nature.

[28] Mills AA, Zheng B, Wang XJ, Vogel H, Roop DR, Bradley A (1999). p63 is a p53 homologue required for limb and epidermal morphogenesis. Nature.

[29] Kouwenhoven EN, van Heeringen SJ, Tena JJ, Oti M, Dutilh BE, Alonso ME (2010). Genome-wide profiling of p63 DNA-binding sites identifies an element that regulates gene expression during limb development in the 7q21 SHFM1 locus. PLoS Genet.

[30] Qu J, Tanis SEJ, Smits JPH, Kouwenhoven EN, Oti M, van den Bogaard EH (2018). Mutant p63 affects epidermal cell identity through rewiring the enhancer landscape. Cell Rep.

[31] Soares E, Xu Q, Li Q, Qu J, Zheng Y, Raeven HHM (2019). Single-cell RNA-seq identifies a reversible mesodermal activation in abnormally specified epithelia of p63 EEC syndrome. Proc Natl Acad Sci USA.

[32] Kouwenhoven EN, van Bokhoven H, Zhou H (2015). Gene regulatory mechanisms orchestrated by p63 in epithelial development and related disorders. Biochim Biophys Acta.

[33] Yang A, Zhu Z, Kapranov P, McKeon F, Church GM, Gingeras TR (2006). Relationships between p63 binding, DNA sequence, transcription activity, and biological function in human cells. Mol Cell.

[34] Yang A, Zhu Z, Kettenbach A, Kapranov P, McKeon F, Gingeras TR (2010). Genome-wide mapping indicates that p73 and p63 co-occupy target sites and have similar dna-binding profiles in vivo. PLoS ONE.

[35] Celli J, Duijf P, Hamel BC, Bamshad M, Kramer B, Smits AP (1999). Heterozygous germline mutations in the p53 homolog p63 are the cause of EEC syndrome. Cell.

[36] Rinne T, Bolat E, Meijer R, Scheffer H, van Bokhoven H (2009). Spectrum of p63 mutations in a selected patient cohort affected with ankyloblepharon-ectodermal defects-cleft lip/palate syndrome (AEC). Am J Med Genet.

[37] Osterburg C, Osterburg S, Zhou H, Missero C, Dotsch V. Isoform-specific roles of mutant p63 in human diseases. Cancers (Basel). 2021;13:536–54. 10.3390/cancers13030536 PMC7866788 33572532

[38] Riege K, Kretzmer H, Sahm A, McDade SS, Hoffmann S, Fischer M. Dissecting the DNA binding landscape and gene regulatory network of p63 and p53. Elife. 2020;9:e63266. 10.7554/eLife.63266 PMC7735755 33263276

[39] Au-Yeung N, Mandhana R, Horvath CM (2013). Transcriptional regulation by STAT1 and STAT2 in the interferon JAK-STAT pathway. JAKSTAT.

[40] Bromberg JF (2001). Activation of STAT proteins and growth control. Bioessays.

[41] Kharbanda S, Ren RB, Pandey P, Shafman TD, Feller SM, Weichselbaum RR (1995). Activation of the C-Abl tyrosine kinase in the stress-response to DNA-damaging agents. Nature.

[42] Kharbanda S, Yuan ZM, Weichselbaum R, Kufe D (1998). Determination of cell fate by c-Abl activation in the response to DNA damage. Oncogene.

[43] Gonfloni S, Di Tella L, Caldarola S, Cannata SM, Klinger FG, Di Bartolomeo C (2009). Inhibition of the c-Abl-TAp63 pathway protects mouse oocytes from chemotherapy-induced death. Nat Med.

[44] Kim SY, Nair DM, Romero M, Serna VA, Koleske AJ, Woodruff TK (2019). Transient inhibition of p53 homologs protects ovarian function from two distinct apoptotic pathways triggered by anticancer therapies. Cell Death Differ.

[45] Grondahl ML, Borup R, Vikesa J, Ernst E, Andersen CY, Lykke-Hartmann K (2013). The dormant and the fully competent oocyte: comparing the transcriptome of human oocytes from primordial follicles and in metaphase II. Mol Hum Reprod.

[46] Adhikari D, Liu K (2009). Molecular mechanisms underlying the activation of mammalian primordial follicles. Endocr Rev.

[47] Maidarti M, Anderson RA, Telfer EE. Crosstalk between PTEN/PI3K/Akt signalling and DNA damage in the oocyte: implications for primordial follicle activation, oocyte quality and ageing. Cells. 2020;9:200–25. 10.3390/cells9010200 PMC7016612 31947601

[48] Castrillon DH, Miao LL, Kollipara R, Horner JW, DePinho RA (2003). Suppression of ovarian follicle activation in mice by the transcription factor Foxo3a. Science.

[49] Arden KC, Biggs WH (2002). Regulation of the FoxO family of transcription factors by phosphatidylinositol-3 kinase-activated signaling. Arch Biochem Biophys.

[50] Liu L, Rajareddy S, Reddy P, Du C, Jagarlamudi K, Shen Y (2007). Infertility caused by retardation of follicular development in mice with oocyte-specific expression of Foxo3a. Development.

[51] Shin I, Rotty J, Wu FY, Arteaga CL (2005). Phosphorylation of p27Kip1 at Thr-157 interferes with its association with importin alpha during G1 and prevents nuclear re-entry. J Biol Chem.

[52] Viglietto G, Motti ML, Bruni P, Melillo RM, D’Alessio A, Califano D (2002). Cytoplasmic relocalization and inhibition of the cyclin-dependent kinase inhibitor p27(Kip1) by PKB/Akt-mediated phosphorylation in breast cancer. Nat Med.

[53] Yang Q, Guan KL (2007). Expanding mTOR signaling. Cell Res.

[54] Adhikari D, Zheng W, Shen Y, Gorre N, Hamalainen T, Cooney AJ (2010). Tsc/mTORC1 signaling in oocytes governs the quiescence and activation of primordial follicles. Hum Mol Genet.

[55] Titus S, Szymanska KJ, Musul B, Turan V, Taylan E, Garcia-Milian R (2021). Individual-oocyte transcriptomic analysis shows that genotoxic chemotherapy depletes human primordial follicle reserve in vivo by triggering proapoptotic pathways without growth activation. Sci Rep.

[56] Christmann M, Kaina B (2013). Transcriptional regulation of human DNA repair genes following genotoxic stress: trigger mechanisms, inducible responses and genotoxic adaptation. Nucleic Acids Res.

[57] Stringer JM, Winship A, Zerafa N, Wakefield M, Hutt K (2020). Oocytes can efficiently repair DNA double-strand breaks to restore genetic integrity and protect offspring health. Proc Natl Acad Sci USA.

[58] Nguyen QN, Zerafa N, Findlay JK, Hickey M, Hutt KJ. DNA repair in primordial follicle oocytes following cisplatin treatment. J Assist Reprod Genet. 2021;38:1405–17. 10.1007/s10815-021-02184-3 PMC8266985 33864208

[59] Dzimianski JV, Scholte FEM, Bergeron E, Pegan SD (2019). ISG15: it’s complicated. J Mol Biol.

[60] Villarroya-Beltri C, Guerra S, Sanchez-Madrid F (2017). ISGylation—a key to lock the cell gates for preventing the spread of threats. J Cell Sci.

[61] Liu H, Cui Y, Bai Y, Fang Y, Gao T, Wang G (2021). The tyrosine kinase c-Abl potentiates interferon-mediated antiviral immunity by STAT1 phosphorylation. iScience.

[62] Kerr JB, Hutt KJ, Cook M, Speed TP, Strasser A, Findlay JK (2012). Cisplatin-induced primordial follicle oocyte killing and loss of fertility are not prevented by imatinib. Nat Med.

[63] Luh LM, Kehrloesser S, Deutsch GB, Gebel J, Coutandin D, Schafer B (2013). Analysis of the oligomeric state and transactivation potential of TAp73alpha. Cell Death Differ.

[64] Agami R, Blandino G, Oren M, Shaul Y (1999). Interaction of c-Abl and p73alpha and their collaboration to induce apoptosis. Nature.

[65] Gong JG, Costanzo A, Yang HQ, Melino G, Kaelin WG, Levrero M (1999). The tyrosine kinase c-Abl regulates p73 in apoptotic response to cisplatin-induced DNA damage. Nature.

[66] Yuan ZM, Shioya H, Ishiko T, Sun X, Gu J, Huang YY (1999). p73 is regulated by tyrosine kinase c-Abl in the apoptotic response to DNA damage. Nature.

[67] Perng YC, Lenschow DJ (2018). ISG15 in antiviral immunity and beyond. Nat Rev Microbiol.

[68] Rossi V, Lispi M, Longobardi S, Mattei M, Di Rella F, Salustri A (2017). LH prevents cisplatin-induced apoptosis in oocytes and preserves female fertility in mouse. Cell Death Differ.

