# Supplementary material for: Enhanced pro-apoptosis gene signature following the activation of TAp63α in oocytes upon γ irradiation
Source: Cell Death Dis. 2022 Mar 4;13(3):204. doi: 10.1038/s41419-022-04659-2 (PMC8897389; doi:10.1038/s41419-022-04659-2)
Supplement: Supplementary file 1 — Supplementary Figures 1-3 [file 41419_2022_4659_MOESM1_ESM.pdf]

Supplementary Figure 1

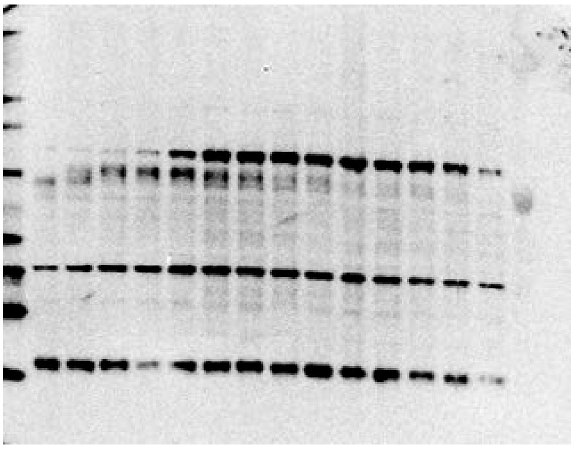

TAp63a and p-TAp63a

Figure 1

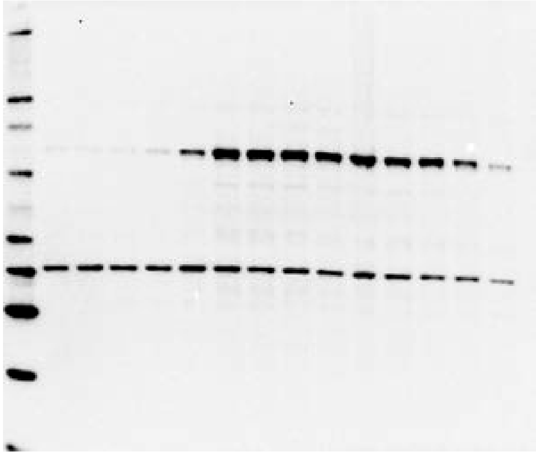

Cleaved PARP1 and Msy2

Figure 1

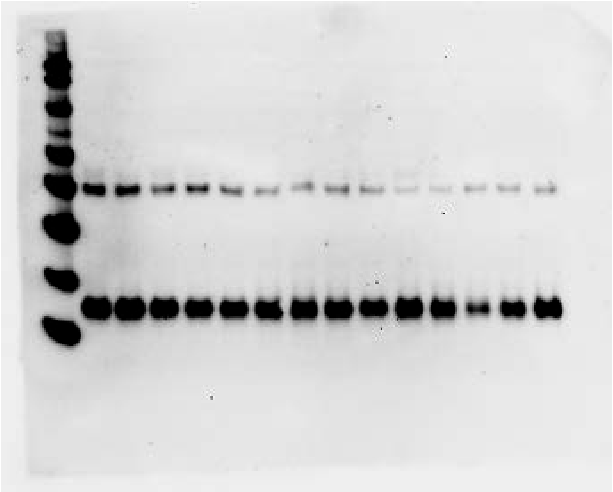

Pro-caspase 9

Figure 1

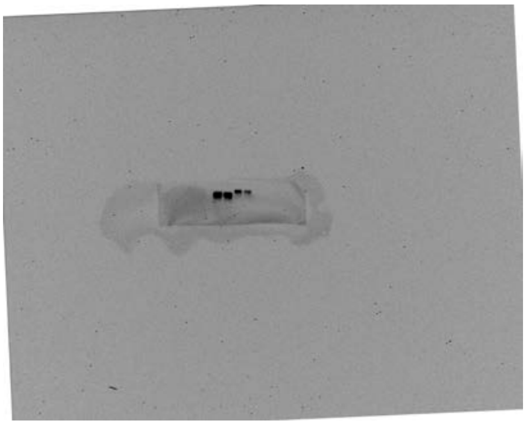

TAp63a

Figure 5b

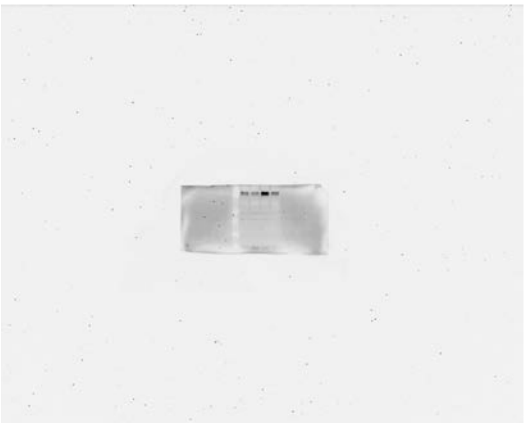

p-STAT1

Figure 5b

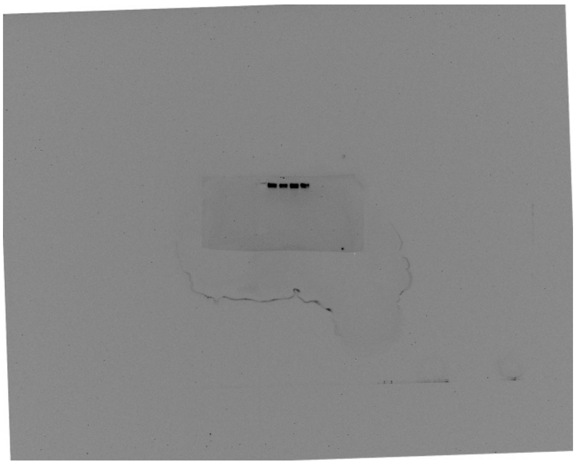

STAT1

Figure 5b

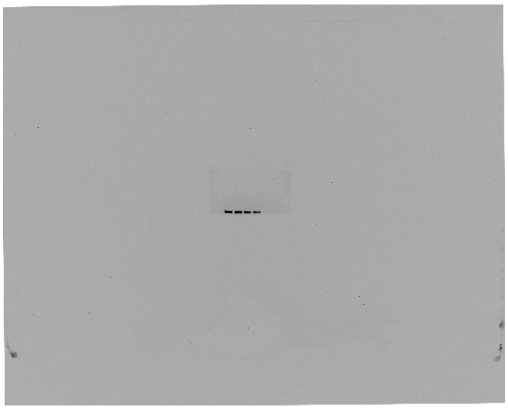

Vinculin

Figure 5b

Supplementary Figure 2

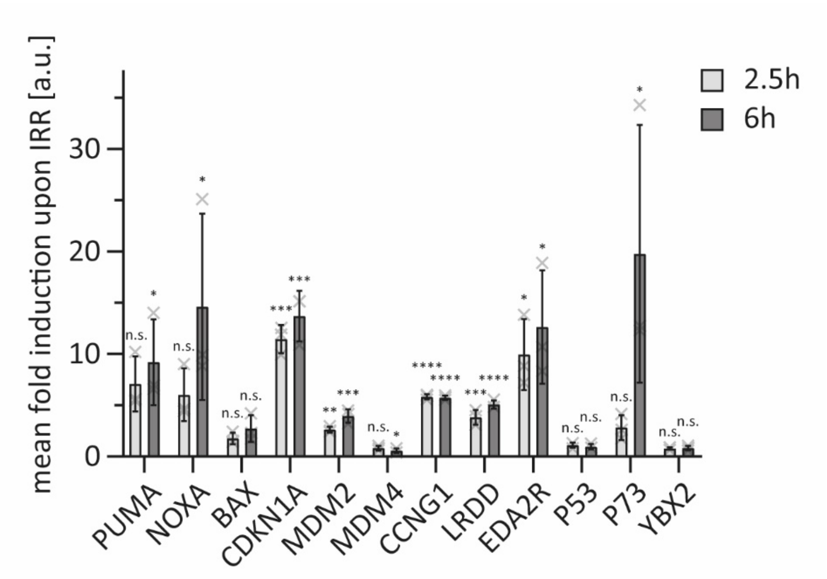

Supplementary Figure 2 .qPCR analysis of selected genes. The fold induction of the time points at 2.5 h (light grey) and 6h (dark grey) relative to the time point before irradiation (0h) is shown. Experiments were conducted in triplicate and data are presented as mean and standard deviation. For each condition per set four dissected ovaries were pooled.

Supplementary Figure 3

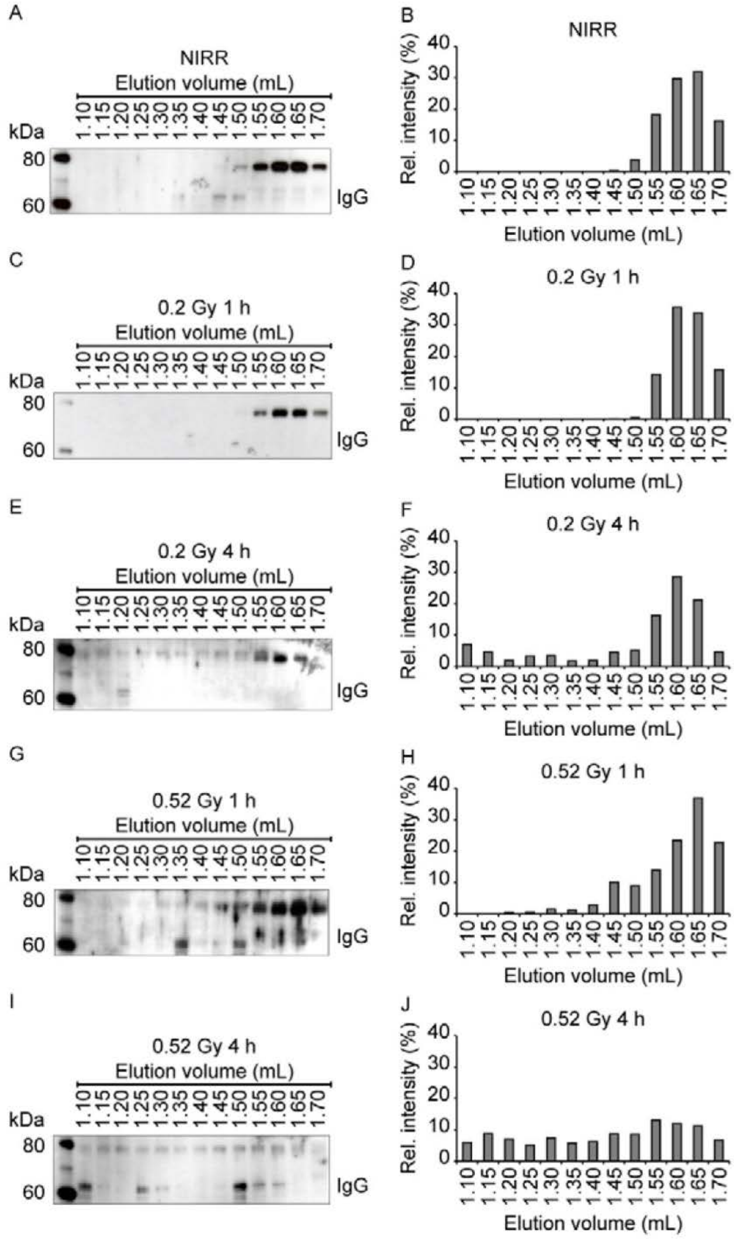

Supplementary Figure 3 Same data as in Figure 7, only including the Western Blot analysis of the size exclusion chromatography runs in addition.
